# Retraction Note: Long noncoding RNA ANRIL is activated by hypoxia-inducible factor-1α and promotes osteosarcoma cell invasion and suppresses cell apoptosis upon hypoxia

**DOI:** 10.1186/s12935-017-0425-7

**Published:** 2017-06-02

**Authors:** Xiaokang Wei, Chuanshun Wang, Chunhui Ma, Wei Sun, Haoqing Li, Zhendong Cai

**Affiliations:** Department of Orthopaedics, Shanghai General Hospital, Shanghai Jiao Tong University School of Medicine, No 100 Hanning Road, Shanghai, 200080 People’s Republic of China

## Retraction to: Cancer Cell Int (2016) 16:73 DOI 10.1186/s12935-016-0349-7

This article [1] has been retracted by the Editor. Figure 2d and the HIF-1a and beta-actin panels (MNNG cell line) shown in Fig. 3c have been duplicated from Zhou et al. [2]. Figure 5a, b have been duplicated from Ma et al. [3]. An investigation by the Academic Ethics Committee of Shanghai General Hospital confirmed that several of the images in this article had been reproduced from previously published articles. In light of this, the results of this study are unreliable. All of the authors agree with this retraction.
